# The evolution of honest and dishonest signals of fighting ability

**DOI:** 10.1093/evlett/qrae008

**Published:** 2024-03-08

**Authors:** Mohammadali Dashtbali, Xiaoyan Long, Jonathan M Henshaw

**Affiliations:** Institute of Biology I, University of Freiburg, Freiburg, Germany; Institute of Biology I, University of Freiburg, Freiburg, Germany; Institute of Biology I, University of Freiburg, Freiburg, Germany

**Keywords:** adaptive dynamics, competition, sexual selection, signaling, causal inference

## Abstract

Competition over resources is often decided via aggressive interactions, which may or may not escalate to all-out fights. Weapons and body size play important roles in such interactions, as they often provide reliable cues of an individual’s fighting ability. In contrast, traits like nonfunctional display “weapons” may dishonestly exaggerate fighting ability in order to intimidate opponents into retreating. Signals used in the context of aggressive interactions potentially evolve via very different mechanisms than courtship signals, but have received far less theoretical attention. Here, we contrast the evolution of honest and dishonest signals of fighting ability using a game-theoretic model. Contests are assumed to consist of three discrete stages: display from a distance, low-intensity physical contact, and fighting. At each stage, contestants evaluate the fighting ability of their opponents in comparison to their own based on body size and an aggressive signal. After making this evaluation, contestants decide whether to escalate the interaction or cede to their opponent. Our model predicts that both honest and dishonest aggressive signals can exaggerate far beyond their ecological optima, but that exaggeration is more pronounced for honest signals. Equilibrium levels of aggressiveness—as measured by individuals’ propensity to escalate aggressive interactions to the next stage—are independent of the honesty of signals. We additionally develop a novel approach, based on causal inference theory, to understand how changes in underlying parameters shape the coevolution of multiple traits. We use this approach to study how aggression coevolves with body and signal size in response to changes in the cost of losing a fight.

## Introduction

Competition over resources is ubiquitous in the natural world. Many of the most spectacular examples involve competition among males for access to females (Andersson, 1994; Emlen, 2008). Traits involved in such competition are often under strong selection and can be as complex and elaborate as those targeted by female preferences (Emlen, 2008; McCullough et al., 2016; O’Brien et al., 2018). Physical confrontations are generally costly due to the risk of injury or predation, as well as the expenditure of energy and time (Arnott and Elwood, 2009; Briffa & Elwood, 2004; Kelly & Godin, 2001). To maximize their fitness, individuals should therefore engage in contests that they are likely to win and avoid contests that they are likely to lose. This, in turn, requires that individuals evaluate their fighting ability (also known as “resource-holding potential”) relative to that of their opponent (Arnott & Elwood, 2008; Jacobs & Heinze, 2017; Parker, 1974). Such evaluations often take place in the context of ritualized agonistic interactions (e.g., threat displays, low-intensity physical contact), in which individuals face far lower risks of injury and death than during physical fights (Andersson, 1994; Gross, 1996). For instance, cichlid fish *Nannacara anomala* engage in tail-beating and swim in parallel orientation (Enquist and Jakobsson, 1986) and red deer *Cervus elaphus* roar and walk side by side (Clutton-Brock & Albon, 1979).

Traits such as body size and weaponry often play a decisive role in fights and may consequently serve as reliable cues of an individual’s fighting ability (Álvarez et al., 2017; Eldred et al., 2016; Hill, 1991; Yasuda and Koga, 2016). In contrast, other traits may dishonestly exaggerate an individual’s fighting ability in order to intimidate opponents into retreating. For instance, many land vertebrates exaggerate their body size during agonistic interactions by raising their fur, feathers, frills, or other body parts (Darwin, 1872; Shine, 1990). In the fiddler crab genus *Uca*, males of some species replace lost claws with lighter, less robust “leptochelous” claws that resemble typical claws but are less effective as weapons (Lailvaux et al., 2009). Such dishonest signals are often physiologically costly to produce. At a social level, they may carry both benefits (e.g., by intimidating potential rivals into retreating) and costs (e.g., by increasing the risk of fights with opponents of superior fighting ability). How these signals coevolve with decision rules for escalating or retreating from aggressive interactions is a fascinating and underexplored question.

Game-theoretic models have greatly contributed to our understanding of animal contests, in particular by characterizing ritualized aggression as an information-gathering process (Enquist & Leimar, 1983; Hurd & Ydenberg, 1996; Maynard Smith & Parker, 1976; Uehara et al., 2007). Most such models focus on optimal decision making when information is acquired cumulatively during agonistic interactions (Eberhard et al., 2018; Enquist & Leimar, 1983; Lorenz, 1963). Fewer models have explored the evolution of aggressive signals themselves. Recently, Moore et al. (2022) provided a quantitative genetic model of sexual selection on a signaling trait that mediates male–male aggression during contests. They argued that signals of aggression can become exaggerated in a runaway process analogous to that acting on sexually selected ornaments (Henshaw & Jones, 2020; Kirkpatrick, 1982; Lande, 1981; Waffender & Henshaw, 2023). However, Moore et al. (2022) did not explicitly model the process of aggressive interactions, nor how individual fighting ability shapes the outcomes of interactions that terminate in physical fights. In particular, body size plays no causal role in their model, but rather evolves solely due to its genetic correlation with the aggressive signal. Signals are thus conceptually divorced from the traits that they are supposedly signaling. For this reason, it remains open how aggressive signals can become exaggerated when expressed alongside other, potentially reliable, cues of fighting ability, such as body size.

In this study, we present a multistage model of dyadic contests in which each contestant can assess their opponent’s fighting ability before deciding whether to escalate or yield to their opponent. Fighting ability is primarily determined by body size. In addition, we consider two scenarios for the relationship between aggressive signals and fighting ability: Such signals are either honest and directly contribute to fighting ability (e.g., weapons) or dishonest and only serve to intimidate opponents. This allows us to contrast the evolution of honest and dishonest signals of fighting ability. We frame our model in terms of males competing over access to a female. However, the model itself is general and could be applied to any situation in which two individuals compete over a resource.

## Materials and methods

**Figure 1:**
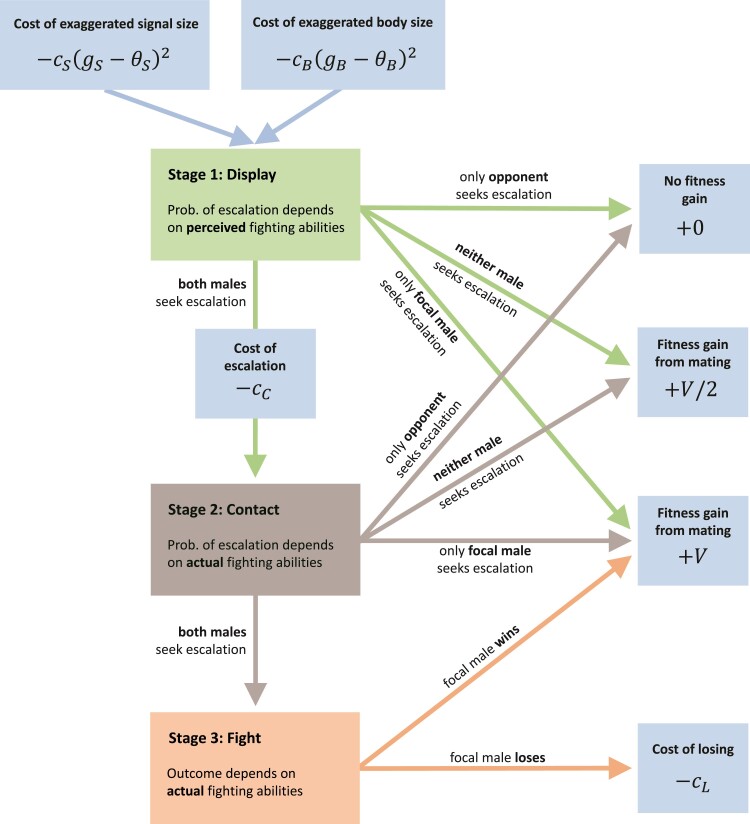
Schematic structure of an aggressive interaction in our model. Blue boxes indicate fitness gains and losses to the focal male.

We model contests between two males over a mating opportunity with a female. A contest consists of three discrete stages in our model (Figure 1): display from a distance (Stage 1), low-intensity physical contact (Stage 2), and fighting (Stage 3). Before the interaction begins, the two males are unfamiliar with each other’s fighting abilities. Displays and physical contact provide both males with information about their relative fighting abilities. Based on this information, each male must decide whether to escalate the conflict to the next stage or yield to his opponent. We assume that the interaction escalates to the next stage only if both parties seek escalation. If one male seeks to escalate, while the other yields, then the escalating individual wins the interaction and mates with the female. If both males yield, then both mate with the female and paternity is shared between them. If the interaction escalates all the way to a fight, then the winner is determined by the males’ relative fighting abilities and the loser pays a cost (e.g., due to injury).

Using an adaptive-dynamics approach (Brännström et al., 2013; Doebeli, 2012), we model the evolution of four quantitative traits: body size B, an aggressive signal S, and an individual’s general tendency to escalate interactions to physical contact $\alpha_{C}$ and to fights $\alpha_{F}$ (see Table 1 for a summary of parameters and variables). The aggressive signal either contributes directly to an individual’s fighting ability (e.g., a weapon) or it merely exaggerates the individual’s *perceived* fighting ability but has no value in a fight. For body size and the aggressive signal, we assume that there are ecological optima (i.e., optimum trait values in the absence of sexual selection) and that deviations from these optima are costly.

**Table 1:** Summary of parameters and variables.

Parameter	Description	Default value
V	Value of resource	0.7
$\delta_{C}$	Extent to which perceived fighting ability influences decision to escalate to physical contact	0.5
$\delta_{F}$	Extent to which actual fighting ability influences decision to escalate to full fight	1
$\delta_{W}$	Extent to which actual fighting ability influences the outcome of fight	1
$\theta_{B}$	Ecologically optimal body size	1.5
$\theta_{S}$	Ecologically optimal signal size	0
$c_{L}$	Cost of losing a fight	1
$c_{S}$	Cost coefficient for deviating from ecologically optimal signal size	0.03
$c_{B}$	Cost coefficient for deviating from ecologically optimal body size	0.15
$c_{C}$	Fixed cost of escalating to physical contact	0.3
$r_{B}$	Extent of environmentally induced variation in body size	0.4
$r_{S}$	Extent of environmentally induced variation in signal size	0.1
Variable	Description	Default initial value
B	Body size	
S	Signal size	
$g_{B}$	Genetic effect on body size	1.5
$g_{S}$	Genetic effect on signal size	0.1
$\alpha_{C}$	Tendency to escalate interactions to physical contact	0.1
$\alpha_{F}$	Tendency to escalate interactions to a full fight	0.1

### Determinants of body and signal size

Both body size B and signal size S are assumed to be determined by the sum of genetic and environmental effects:


$$\begin{array}{c} B=g_{B}+e_{B}\\ S=g_{S}+e_{S}. \end{array}$$ (1)

We follow the standard adaptive-dynamics assumption that new mutations arise against a backdrop of a genetically uniform population. Thus, all individuals in the population, with the exception of rare mutants, carry the same genetic values $g_{B}$ and $g_{S}$. Variation in body and signal size is nonetheless maintained by the environmental effects in our model. This ensures, in particular, that individual males do not know the true body size or signal size of their opponents in advance.

For convenience, we assume that environmental effects $e_{B}$ and $e_{S}$ are independent and uniformly distributed, according to:


$$\begin{array}{c} e_{B}∼𝒰\,\left(-r_{B}\,g_{B},r_{B}\,g_{B}\right)\\ e_{S}∼𝒰\,\left(-r_{S}\,g_{S},r_{S}\,g_{S}\right). \end{array}$$ (2)

The parameters $r_{B}$ and $r_{S}$ determine the extent of environmentally induced variation in body size and signal size, respectively. Under these assumptions, the realized body size and signal size of an individual with genetic values $g_{B}$ and $g_{S}$ are independent, with distributions given by:


$$\begin{array}{c} B∼𝒰\,\left(\left(1-r_{B}\right)\,g_{B},\left(1+r_{B}\right)\,g_{B}\right)\\ S∼𝒰\,\left(\left(1-r_{S}\right)\,g_{S},\left(1+r_{S}\right)\,g_{S}\right). \end{array}$$ (3)

Note that the standard deviation of each trait is assumed proportional to the mean value (i.e., the coefficients of variation are constant). We chose uniform distributions for mathematical tractability, but do not expect that our qualitative conclusions would differ under a more biologically realistic choice of distribution.

### Actual and perceived fighting ability

**Figure 2:**
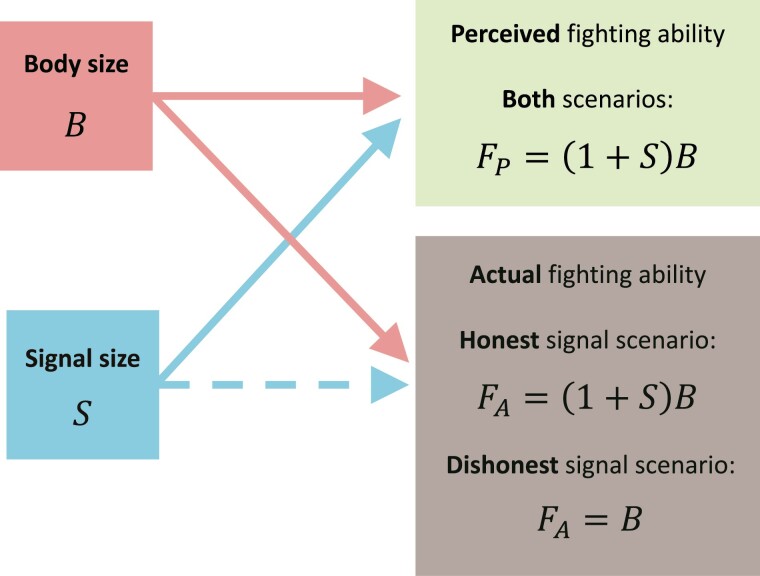
Relationships between body size, signal size, and fighting ability. In the honest signal scenario, fighting ability—whether perceived or actual—is an increasing function of both body size and signal size. In the dishonest signal scenario, actual fighting ability depends on body size alone, but perceived fighting ability additionally increases with signal size.

**Figure 3:**
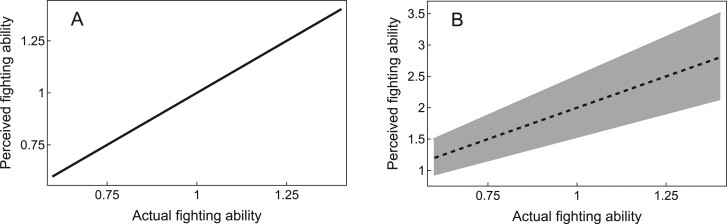
Relationship between perceived fighting ability and actual fighting ability when (A) the aggressive signal contributes equally to both actual and perceived fighting ability (honest signal scenario) and (B) the aggressive signal exaggerates perceived fighting ability but does not contribute to actual fighting ability (dishonest signal scenario). Under the dishonest signal scenario, there is no one-to-one correspondence between perceived and actual fighting ability (denoted by the gray region). Shown with $g_{S}=1$ and $r_{S}=0.5.$

Body size is the primary determinant of a male’s fighting ability. We consider two possible scenarios (Figure 2) for the relationship between a male’s aggressive signal and his fighting ability (Figure 3):

1. **Dishonest signal scenario**: In this scenario, the aggressive signal increases a male’s *perceived* fighting ability $F_{P}$ in the eyes of his opponent, but does not increase his *actual* fighting ability $F_{A}$. Perceived fighting ability increases with both body size and the aggressive signal, according to:
  $$F_{P}=\left(1+S\right)\,B.$$ (4)
  In contrast, actual fighting ability is determined by body size alone:
  $$F_{A}=B.$$ (5)
  In this case, the aggressive signal can be viewed as a dishonest exaggeration of a male’s fighting ability. It may function to intimidate rivals, but has no value in a fight. Display traits that exaggerate an individual’s perceived body size, such as erect body posture, low-pitch vocalizations, or raised fur or feathers fall under this scenario.
2. **Honest signal scenario**: Here, the aggressive signal increases both a male’s perceived fighting ability $F_{P}$ and his actual fighting ability $F_{A}$:
  $$F_{P}=F_{A}=\left(1+S\right)\,B.$$ (6)
  In this case, the signal contributes directly to a male’s fighting ability and so can be viewed as an honest signal. Weapons such as antlers, horns, and claws that are employed in fights are examples of such signals (Emlen, 2008).

For simplicity, we assume that individuals do not perceive the body size and signal size of their opponents as separate traits, but rather always in combination via perceived or actual fighting ability. For honest signals, there is no need for individuals to separate these two traits, as they affect perceived and actual fighting ability in the same manner. For dishonest signals, the inability to distinguish true body size contributes to the effectiveness of the signal.

### Costs of body size and signals

In addition to fitness changes resulting from contests, we assume that males pay fixed costs for maintaining body and signal sizes that diverge from their ecological optima. The cost of deviating from the ecologically optimal body size $\theta_{B}$ is given by $c_{B}\,{\left(g_{B}-\theta_{B}\right)}^{2}$. The parameter $c_{B}$ determines how steeply costs increase as males deviate from optimal body size. Similarly, the cost of deviating from the ecologically optimal signal size $\theta_{S}$ (e.g., due to increased predation or energy expenditure) is given by $c_{S}\,{\left(g_{S}-\theta_{S}\right)}^{2}$, where $c_{S}$ controls how steeply costs rise with increasing signal size. The total cost of both traits is then:


$$c\,\left(g_{B},g_{S}\right)=c_{B}\,{\left(g_{B}-\theta_{B}\right)}^{2}+c_{S}\,{\left(g_{S}-\theta_{S}\right)}^{2}.$$ (7)

For convenience, we scale $c_{B}$ and $c_{S}$, so that these terms indicate the average cost per contest (i.e., we divide their raw values by the average number of contests over a male lifetime). We will now consider each stage of the contest in turn.

### Stage 1: Display

**Figure 4:**
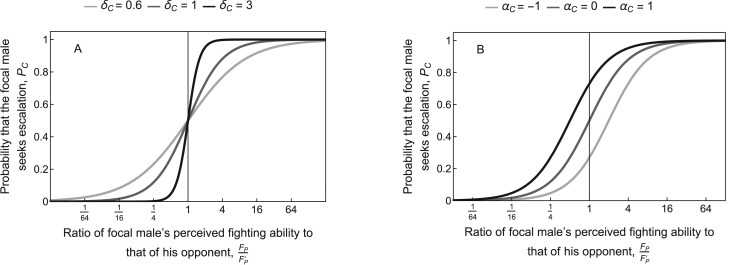
Probability $P_{C}$ that the focal male seeks escalation to physical contact as a function of the ratio $\frac{F_{P}}{F_{P}^{\prime }}$ of the focal male’s perceived fighting ability to that of his opponent. Shown with variation in (A) the informativeness of the display $\delta_{C}$ (all lines with $\alpha_{C}=0$) and (B) the focal male’s general tendency to escalate to physical contact $\alpha_{C}$ (all lines with $\delta_{C}=1$).


Let us now consider a contest between a focal male with trait values $\left(g_{B},g_{S},\alpha_{C},\alpha_{F}\right)$ and an opponent with trait values $\left(g_{B}^{\prime },g_{S}^{\prime },\alpha_{C}^{\prime },\alpha_{F}^{\prime }\right)$ (variables relating to the opponent are marked with a prime ′ throughout). During the first stage, the males display to each other from a distance. These displays provide information about each male’s *perceived* fighting ability. Based on this information, each male decides whether to escalate to physical contact. We write $P_{C}$ for the probability that the focal male seeks escalation and $P_{C}^{\prime }$ for that of his opponent. We assume that $P_{C}$ increases with the ratio $\frac{F_{P}}{F_{P}^{\prime }}$ of the focal male’s perceived fighting ability to that of his opponent, according to the sigmoidal function (Figure 4):

$$P_{C}\,\left(F_{P},F_{P}^{\prime },\alpha_{C}\right)={\left(1+e^{-\alpha_{C}}\,{\left(\frac{F_{P}^{\prime }}{F_{P}}\right)}^{\delta_{C}}\right)}^{-1}.$$ (8)

The probability $P_{C}^{\prime }$ that the opponent seeks escalation is defined analogously. Such a sigmoidal relationship could arise, for example, if individuals perceive the ratio $F_{P}/F_{P}^{\prime }$ with some error and decide to escalate if and only if the perceived ratio exceeds a threshold value (Vickers, 1970). In the above formulation, the threshold is determined by the evolving trait $\alpha_{C}$, such that males with larger $\alpha_{C}$ are more willing to escalate for any given ratio $F_{P}/F_{P}^{\prime }$ of perceived fighting abilities. The parameter $\delta_{C}\geq 0$ determines the extent to which differences in perceived fighting ability influence the decision to escalate, which should depend on the magnitude of noise in perception of $F_{P}/F_{P}^{\prime }$. If $\delta_{C}=0$, then each male seeks escalation with a fixed probability determined only by his own trait $\alpha_{C}$, regardless of his own fighting ability and that of his opponent. If $\delta_{C}$ is large, then the focal male will escalate if the ratio $F_{P}/F_{P}^{\prime }$ exceeds a threshold that is determined by his trait $\alpha_{C}$ (e.g., with $\alpha_{C}=0$, the focal male will escalate whenever his perceived fighting ability is greater than that of his opponent). We can thus interpret the parameter $\delta_{C}$ as the informativeness of the display: Higher $\delta_{C}$ means that males can estimate perceived (but not necessarily actual) fighting ability more precisely.

Analogously to the hawk-dove game (Maynard Smith, 1982; Maynard Smith & Price, 1973), we consider the following outcomes of such a contest (Figure 1):

1. The focal male seeks escalation, but his opponent yields (probability $P_{C}\,\left(1-P_{C}^{\prime }\right)$). In this case, the focal male mates with the female, gaining a fitness benefit of V.
2. The opponent seeks escalation, but the focal male yields (probability $\left(1-P_{C}\right)\,P_{C}^{\prime }$). In this case, the opponent mates with the female and the focal male’s fitness is unchanged.
3. Both males yield (probability $\left(1-P_{C}\right)\,\left(1-P_{C}^{\prime }\right)$). In this case, both males mate with the female and the fitness benefit of siring her offspring is shared equally between them, leading to a fitness benefit of $\frac{V}{2}$ for the focal male.
4. Both males seek escalation (probability $P_{C}\,P_{C}^{\prime }$). In this case, the interaction escalates to physical contact. The focal males’ expected fitness gain in the next stage is given by $E\,\left(\Delta \,W_{2}\right)$ (see next section).

For fixed trait values, the fitness gain to the focal male is thus given by:


$$\begin{array}{cc} \Delta \,W_{1} & =-c\,\left(g_{B},g_{S}\right)+P_{C}\,\left(1-P_{C}^{\prime }\right)\,V+\left(1-P_{C}\right)\,\left(1-P_{C}^{\prime }\right)\,\frac{V}{2}\\ & +P_{C}\,P_{C}^{\prime }\,E\,\left(\Delta \,W_{2}\right). \end{array}$$ (9)

Integrating over the trait distribution of the focal and opponent males, we thus have


$$E\,\left(\Delta \,W_{1}\right)=\int_{\mathrm{traits}}f_{1}\,\Delta \,W_{1},$$ (10)

where $\int_{\mathrm{traits}}$ integrates over all trait values:


$$\begin{array}{cc} \int_{\mathrm{traits}} & =\int_{\left(1-r_{S}\right)\,g_{S}^{\prime }}^{\left(1+r_{S}\right)\,g_{S}^{\prime }}\int_{\left(1-r_{S}\right)\,g_{S}}^{\left(1+r_{S}\right)\,g_{S}}\int_{\left(1-r_{B}\right)\,g_{B}^{\prime }}^{\left(1+r_{B}\right)\,g_{B}^{\prime }}\int_{\left(1-r_{B}\right)\,g_{B}}^{\left(1+r_{B}\right)\,g_{B}}\\ & d\,B\,d\,B^{\prime }\,d\,S\,d\,S^{\prime } \end{array}$$ (11)

and $f_{1}$ is the probability density function for the joint distribution of all traits:


f1=116⁢rB2⁢rS2⁢gB⁢gB′⁢gS⁢gS′. (12)

### Stage 2: Contact

If both males seek escalation in Stage 1, then the interaction proceeds to low-intensity physical contact. Such contact carries a risk of injury, which we represent as a small fixed cost $c_{C}$ of escalating to this stage for both males. During contact, males gain information about their (relative) *actual* fighting abilities. We assume that the focal male’s probability $P_{F}$ of seeking escalation to a full fight is determined by the ratio of actual fighting abilities, according to:


$$P_{F}\,\left(F_{A},F_{A}^{\prime },\alpha_{F}\right)={\left(1+e^{-\alpha_{F}}\,{\left(\frac{F_{A}^{\prime }}{F_{A}}\right)}^{\delta_{F}}\right)}^{-1}.$$ (13)

Analogous to Equation (8) above, $\delta_{F}\geq 0$ determines how much information about relative fighting ability is gained via physical contact, while $\alpha_{F}$ is the focal male’s overall tendency to seek escalation to a fight. In our model, we assume that estimates of actual fighting ability in this stage are more precise than estimates of perceived fighting ability in the display stage (i.e., $\delta_{F}>\delta_{C}$). The probability $P_{F}^{\prime }$ that the opponent seeks escalation is defined analogously. Note that, since the probability of escalation depends on actual fighting ability in this stage, this probability depends on both body size and the aggressive signal in the honest signal scenario, but on body size only in the dishonest signal scenario.

The fitness gain to the focal male for fixed trait values is given by:


$$\begin{array}{cc} \Delta \,W_{2} & =-c_{C}+P_{F}\,\left(1-P_{F}^{\prime }\right)\,V+\left(1-P_{F}\right)\,\left(1-P_{F}^{\prime }\right)\,\frac{V}{2}\\ & +P_{F}\,P_{F}^{\prime }\,E\,\left(\Delta \,W_{3}\right). \end{array}$$ (14)

Integrating over the joint distribution of traits then yields:


$$E\,\left(\Delta \,W_{2}\right)=\int_{\mathrm{traits}}f_{2}\,\Delta \,W_{2},$$ (15)

where $f_{2}$ is the joint probability density function of male traits in Stage 2:


$$f_{2}=\frac{f_{1}\,P_{C}\,P_{C}^{\prime }}{\int_{\mathrm{traits}}f_{1}\,P_{C}\,P_{C}^{\prime }}.$$ (16)

Note that this distribution differs from that in the first stage, because only a subset of males escalates to the second stage.

### Stage 3: Fight

If both males seek escalation in Stage 2, then a full fight occurs. The probability $P_{W}$ that the focal male wins the fight depends on the ratio of the males’ actual fighting abilities, according to:


$$P_{W}\,\left(F_{A},F_{A}^{\prime }\right)={\left(1+{\left(\frac{F_{A}^{\prime }}{F_{A}}\right)}^{\delta_{W}}\right)}^{-1}.$$ (17)

The parameter $\delta_{W}$ determines how sensitive the outcomes of fights are to differences in fighting ability. If $\delta_{W}=0$, then each male wins the fight with a probability of 0.5, regardless of fighting ability. If $\delta_{W}$ is very large, then the better fighter always wins. The probability $P_{W}^{\prime }$ that the opponent wins is defined symmetrically (note that $P_{W}+P_{W}^{\prime }=1$).

The focal male gains fitness of V if he wins the fight, but suffers a fitness loss of $c_{L}$ if he loses. For simplicity, we do not incorporate a cost of injury to the winner of the fight (which would result in a reduced value of V in the final stage), but rather maintain the same value of V as in the previous stages. The focal male’s fitness change in Stage 3 is therefore:


$$\Delta \,W_{3}=P_{W}\,V-\left(1-P_{W}\right)\,c_{L}.$$ (18)

Integrating over the joint distribution of all traits, we have:


$$E\,\left(\Delta \,W_{3}\right)=\int_{\mathrm{traits}}f_{3}\,\Delta \,W_{3},$$ (19)

where $f_{3}$ is the joint probability density of all traits in Stage 3:


$$f_{3}=\frac{f_{2}\,P_{F}\,P_{F}^{\prime }}{\int_{\mathrm{traits}}f_{2}\,P_{F}\,P_{F}^{\prime }}.$$ (20)

### Evolutionary equilibrium

Consider now a mutant with trait values $\left(g_{B},g_{S},\alpha_{C},\alpha_{F}\right)$ in a population where all other individuals (in particular, all potential opponents of the mutant) have trait values $\left(g_{B}^{\prime },g_{S}^{\prime },\alpha_{C}^{\prime },\alpha_{F}^{\prime }\right)$. The selection gradient acting on mutant trait values is given by:


$$\beta ={\left(\begin{array}{c} \frac{\partial E\,\left(\Delta \,W_{1}\right)}{\partial g_{B}}\\ \frac{\partial E\,\left(\Delta \,W_{1}\right)}{\partial g_{S}}\\ \frac{\partial E\,\left(\Delta \,W_{1}\right)}{\partial \alpha_{C}}\\ \frac{\partial E\,\left(\Delta \,W_{1}\right)}{\partial \alpha_{F}} \end{array}\right)|}_{\begin{array}{c} g_{B}=g_{B}^{\prime },g_{S}=g_{S}^{\prime },\\ \alpha_{C}=\alpha_{C}^{\prime },\alpha_{F}=\alpha_{F}^{\prime } \end{array}}.$$ (21)

In practice, we approximated selection gradients numerically using the rule $f^{\prime }\,\left(x\right)\approx \frac{1}{2\,\varepsilon }\,\left(f\,\left(x+\varepsilon \right)-f\,\left(x-\varepsilon \right)\right)$ with ε=0.001. All integrals in the fitness function were evaluated numerically using Wolfram Mathematica’s NIntegrate[] function (see Mathematica code in Supplementary Materials).

Starting with arbitrary initial strategies $\left(g_{B0},g_{S0},\alpha_{C0},\alpha_{F0}\right)$, we followed the selection trajectories given by the selection gradients β numerically until the strategies converged to a state of evolutionary equilibrium. This was accomplished by iterating the following equation (Johns et al., 2019):


(gBm+1gSm+1αCm+1αFm+1)=(gBmgSmαCmαFm)+q⁢β, (22)

where q is a small positive constant (we found q=0.5 suitable; see Mathematica code in Supplementary Materials). In other words, the strategies take a small step in the direction of selection at each time step, with the magnitude of the step proportional to the intensity of selection. Different starting strategies always resulted in the same equilibrium.

## Results

### Honest signals were larger than dishonest signals

**Figure 5:**
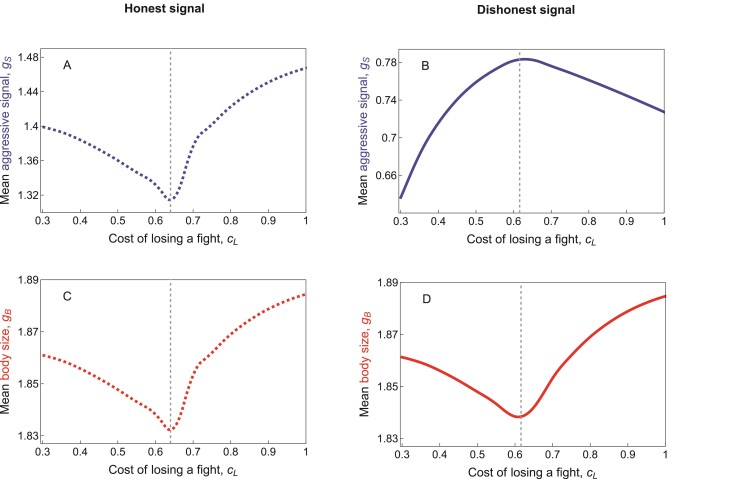
Equilibrium values of mean aggressive signal (blue) and mean body size (red) with respect to the cost of losing a fight $c_{L}$ under the honest signal scenario (dashed lines, (A) and (C)) and the dishonest signal scenario (solid lines, (B) and (D)). Under both the honest and dishonest signal scenarios, equilibrium mean body size shows a roughly V-shaped relationship with the cost of losing a fight. The equilibrium size of honest signals shows a similar relationship. In contrast, dishonest signals show the opposite relationship, increasing with the cost of losing a fight for small values of $c_{L}$ and then decreasing again at larger values. The vertical dashed lines indicate the $c_{L}$ value at which the relationship changes from positive to negative, or vice versa. Honest signals are consistently larger than dishonest signals. Other parameters take their default values (Table 1): In particular, $V=0.7;\theta_{B}=1.5;\theta_{S}=0;\delta_{C}=0.5;\delta_{F}=1;\delta_{W}=1;c_{S}=0.03;c_{C}=0.3$. For $c_{L}>V$, we used initial values as in Table 1. For $c_{L}<V$, convergence was very slow. To speed up convergence in this parameter range, we consequently took the equilibrium trait values for each $c_{L}$-value as initial values for the next-smallest $c_{L}$-value.

Both honest and dishonest signals evolved to exaggerated sizes under a wide range of parameter settings in our model (Figure 5A and B). However, all else being equal, honest signals were consistently larger at equilibrium than dishonest signals (compare the ranges of $g_{S}$ on the vertical axes between Figure 5A and B). This is because honest signals have dual functions, serving both as signals and weapons. Interestingly, the cost of losing a fight $c_{L}$ influenced equilibrium body and signal size in a complex manner that differed qualitatively between honest and dishonest signals (Figure 5). This pattern is shaped by the coevolution of body and signal size with individuals’ willingness to escalate interactions to the next stage; we consequently first consider the evolution of willingness to escalate, before returning to explain how body size and signal size change with the cost of losing a fight. Here, we present verbal explanations for the observed patterns, which are justified via formal causal analyses in Supplementary Materials (see also Supplementary Figure S1).

### Individuals avoided low-value and high-cost fights, with subtle implications for the willingness to make physical contact

The equilibrium tendencies to escalate interactions did not differ substantially between the honest and dishonest signal scenarios. This is because these traits mainly depend on the potential fitness costs and benefits of each decision, which do not depend on signal honesty in our model. We consequently present results for the dishonest signal scenario here; results for the honest signal scenario are presented in Supplementary Materials (see Supplementary Figures S2 and S3).

**Figure 6:**
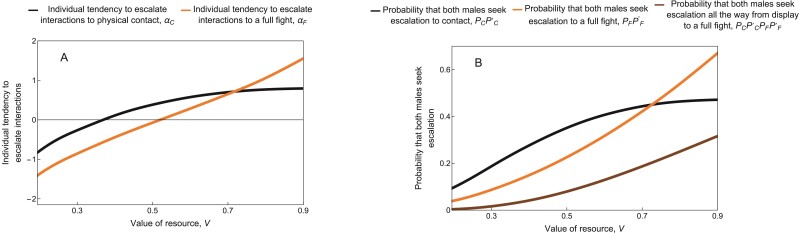
(A) Equilibrium tendency to escalate interactions to physical contact, $\alpha_{C}$ (black) or to a full fight, $\alpha_{F}$ (orange) for the dishonest signal scenario with respect to the value of resource. (B) The probability that a pair of males escalates the interaction from the display stage to physical contact, $P_{C}\,P_{C}^{\prime }$ (black), from the contact stage to a full fight, $P_{F}\,P_{F}^{\prime }$ (orange), or from the display stage all the way to a full fight, $P_{C}\,P_{C}^{\prime }\,P_{F}\,P_{F}^{\prime }$ (brown) for the dishonest signal scenario, averaging over all environmental effects. As the value of resource V increases, individuals are more likely to escalate to physical contact and to a full fight. This results in a greater overall probability of a full fight. Other parameters take their default values (Table 1): In particular, $c_{L}=1;\theta_{B}=1.5;\theta_{S}=0;\delta_{C}=0.5;\delta_{F}=1;\delta_{W}=1;c_{S}=0.03;c_{C}=0.3$.

If the value of the resource V was low relative to the cost of losing a fight $c_{L}$, individuals were less willing to escalate to both physical contact (low $\alpha_{C}$) and to full fights (low $\alpha_{F}$) (Figure 6A, depicted with $V<c_{L}=1$). Consequently, most aggressive interactions were resolved at the display stage (low $P_{C}\,P_{C}^{\prime }$, Figure 6B).

**Figure 7:**
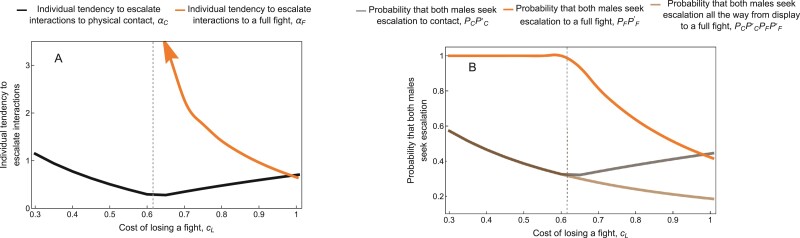
(A) Equilibrium tendency to escalate interactions to physical contact, $\alpha_{C}$ (black) or to a full fight, $\alpha_{F}$ (orange) in the dishonest signal scenario with respect to the cost of losing a fight. (B) The probability that a pair of males escalates the interaction from the display stage to physical contact, $P_{C}\,P_{C}^{\prime }$ (gray), from the contact stage to a full fight, $P_{F}\,P_{F}^{\prime }$ (orange), or from the display stage all the way to a full fight, $P_{C}\,P_{C}^{\prime }\,P_{F}\,P_{F}^{\prime }$ (brown) in the dishonest signal scenario, averaging over all environmental effects. Individuals are more willing to escalate from physical contact to a full fight when $c_{L}$ is small, such that $\alpha_{F}$ approaches infinity as $c_{L}$ becomes smaller (indicated by the orange arrow). Consequently, physical contact always leads directly to a fight when $c_{L}$ is small. In contrast, willingness to escalate from display to physical contact shows a V-shaped relationship with $c_{L}$, first decreasing and then increasing again. The overall probability that an aggressive interaction escalates all the way from the display stage to a full fight nonetheless decreases monotonically with $c_{L}$. The vertical dashed lines correspond to the $c_{L}$ value indicated by the vertical dashed lines in Figure 5B and D. Analogous results for the honest signal scenario are shown in Supplementary Figure S3. Other parameters take their default values (Table 1): in particular, $V=0.7;\theta_{B}=1.5;\theta_{S}=0;\delta_{C}=0.5;\delta_{F}=1;\delta_{W}=1;c_{S}=0.03;c_{C}=0.3.$ For $c_{L}>V$, we used initial values as in Table 1. For $c_{L}<V$, convergence was very slow. To speed up convergence in this parameter range, we consequently took the equilibrium trait values for each $c_{L}$-value as initial values for the next-smallest $c_{L}$-value.

Conversely, when the cost of losing a fight $c_{L}$ was small relative to the benefit V, individuals that had already reached the contact stage always escalated to a full fight (i.e., large $\alpha_{F}$, corresponding to $P_{F}\,P_{F}^{\prime }\approx 1$; see the orange line to the left of the vertical dashed line in Figure 7B). However, due to the additional cost $c_{C}$ of escalating to contact, not all interactions escalated from the display stage to physical contact (i.e., small $\alpha_{C}$, corresponding to small $P_{C}\,P_{C}^{\prime }$; see the gray line to the left of the vertical dashed line in Figure 7B). Individuals escalating from the display stage to contact faced both the certainty of paying $c_{C}$ and an inevitable fight, with the possibility of paying an additional $c_{L}$ if they lost. As a result, the expected fitness gain in the contact stage ($E\,\left(\Delta \,W_{2}\right)$) declined as the cost of losing a fight $c_{L}$ increased. This led to a decreasing willingness to escalate to contact (smaller $\alpha_{C}$) with increasing $c_{L}$ (see the black line to the left of the vertical dashed line in Figure 7A).

The situation was more nuanced at higher values of $c_{L}$ (to the right of the vertical dashed line in Figure 7). In this case, not all cases of physical contact escalated to a full fight. Rather, as the cost of losing a fight increased, individuals were less willing to escalate from physical contact to a full fight (smaller $\alpha_{F}$) and the probability $P_{F}\,P_{F}^{\prime }$ of such escalation was consequently reduced (orange lines to the right of the vertical dashed lines in Figure 7). This pattern is similar to Figure 6A, where $\alpha_{F}$ decreased as the value of the resource V declined.

Surprisingly, however, as the cost of losing a fight rose, individuals *increased* their willingness to escalate from display to contact (larger $\alpha_{C}$) (see the black line to the right of the vertical dashed line in Figure 7A). The increase in $\alpha_{C}$ arose indirectly as a flow-on effect of decreased $\alpha_{F}$. The reason is as follows: With a reduced willingness to escalate from contact to a full fight (smaller $\alpha_{F}$), there was a greater chance of resolving conflicts in the contact stage (i.e, larger $1-P_{F}\,P_{F}^{\prime }$), without needing to engage in a costly fight. This led to a larger expected fitness gain in the contact stage (larger $E\,\left(\Delta \,W_{2}\right)$), selecting for the observed increase in $\alpha_{C}$. As a consequence of these interacting patterns, the overall relationship between the cost of losing a fight ($c_{L}$) and the willingness to escalate to contact ($\alpha_{C}$) was V-shaped (Figure 7A).

It might seem surprising that $\alpha_{C}$ increased when fights were more costly in (the right half of) Figure 7, but decreased when the resource was less valuable in Figure 7. This difference arises because when the resource is less valuable, the fitness gain $V-c_{C}$ for a successful male in the contact stage is reduced, which reduces the overall expected fitness gain in this stage ($E\,\left(\Delta \,W_{2}\right)$). In contrast, increasing the cost of losing only directly reduces the expected fitness gain in the final stage ($E\,\left(\Delta \,W_{3}\right)$). How this reduction in $E\,\left(\Delta \,W_{3}\right)$ translates into a change in $E\,\left(\Delta \,W_{2}\right)$ depends on the population tendency to escalate to a full fight $\alpha_{F}$, however, which is an evolving parameter.

To validate the above verbal interpretations, we conducted causal analyses (see Supplementary Materials). By artificially holding $\alpha_{F}$ fixed, we found that $\alpha_{C}$ continued to decrease with $c_{L}$ even when $c_{L}$ was large (Supplementary Figures S4A and S5A). This is intuitive, as escalation at any stage increases an individual’s risk of losing a fight and paying the associated cost, all else being equal. In our model, therefore, there is a negative direct effect of $c_{L}$ on $\alpha_{C}$, while the indirect effect of $c_{L}$ on $\alpha_{C}$ via $\alpha_{F}$ is positive. When $c_{L}$ is relatively small, the direct effect is more pronounced; however, the indirect effect becomes more important when $c_{L}$ is relatively large.

### The cost of losing influenced body and signal size in complex ways

We can now return to explain how the cost of losing a fight influences the evolution of body size and signal size. Dishonest signals are the most straightforward case (Figure 5B). Since these signals are only useful during displays, their fitness benefits are highest when most interactions are resolved in the display stage, which occurs when the willingness to escalate to contact $\alpha_{C}$ is small. Individuals consequently invested less in dishonest signals as $\alpha_{C}$ increased. The overall relationship of $\alpha_{C}$ with the cost of losing a fight was V-shaped (Figure 7; see explanation above). As a result, the size of dishonest signals showed an inverse V-shaped relationship with the cost of losing, first increasing and then decreasing as $c_{L}$ increased (Figure 5B).

Honest signals and body size had more complex coevolutionary dynamics (Figure 5A, C, and D), since these traits are important in every stage of an aggressive interaction. If willingness to escalate ($\alpha_{C}$ and $\alpha_{F}$) was artificially held fixed, both honest signals and body size increased with the cost of losing a fight (see Causal analyses in Supplementary Materials and Supplementary Figure S5C). This makes sense, since these traits help individuals both to intimidate opponents—and thus avoid fights entirely—and to win fights, thereby avoiding the cost of losing. These traits were consequently more strongly favored when the cost of losing a fight was greater, all else being equal. Furthermore, the optimal values of both traits increased as individuals’ willingness to escalate increased (larger $\alpha_{C}$ or $\alpha_{F}$, see Causal analyses in Supplementary Materials) because more frequent fights mean that the cost of losing is paid more often. In turn, the willingness to escalate fights depended in a complex manner on $c_{L}$ (Figure 7A; see explanation above).

The overall effect of $c_{L}$ on honest signals thus represented an interplay between the direct effect of $c_{L}$ and the indirect effects via $\alpha_{C}$ and $\alpha_{F}$. These pathways interacted as follows: When $c_{L}$ was relatively small (to the left of the vertical dashed lines in Figures 5A, C, D and 7A), the willingness to escalate to contact $\alpha_{C}$ decreased with $c_{L}$, but contact always led to a full fight (large $\alpha_{F}$). Since $\alpha_{F}$ was always large in this parameter range, changes in $c_{L}$ did not affect the other traits via changes in $\alpha_{F}$. On the other hand, decreasing $\alpha_{C}$ had a large effect on equilibrium body size and honest signal size, acting to reduce the size of both traits. Furthermore, this effect overwhelmed the positive direct effect of $c_{L}$, such that these traits did indeed decrease with $c_{L}$. As a result, honest signals and body size deceased with $c_{L}$ as long as $c_{L}$ was relatively small. For larger values of $c_{L}$ (to the right of the vertical dashed line in Figures 5A, C, D and 7A), $\alpha_{C}$ increased, while $\alpha_{F}$ decreased with $c_{L}$. The direct effect of $c_{L}$ and the indirect effect of $c_{L}$ via $\alpha_{C}$ acted in the same direction in this case—to favor increasing the size of both traits—but were opposed by the indirect effect of $c_{L}$ via $\alpha_{F}$. The net result was that body size and honest signal size increased with $c_{L}$ when $c_{L}$ was relatively large. Taken all together, we observed that both honest signals and body size showed a V-shaped relationship with the cost of losing a fight (Figure 5A, C, and D).

### Signal size decreased with signal cost, but increased with signal informativeness

**Figure 8:**
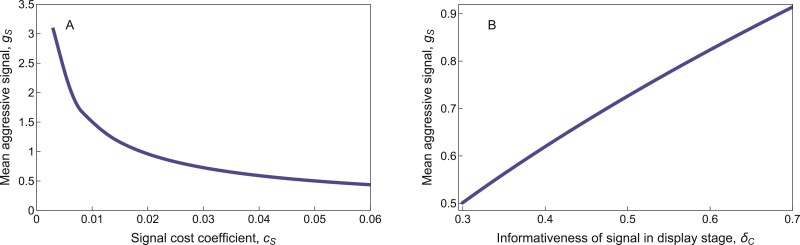
Equilibrium mean signal size with respect to (A) the signal cost coefficient, $c_{S}$ (shown with $\delta_{C}=0.5$), and (B) the informativeness of the signal in the display stage, $\delta_{C}$ (shown with $c_{S}=0.03$) in the dishonest signal scenario. Equilibrium signal size decreases with the cost of the signal, but increases when the signal is a more informative indicator of perceived fighting ability. Analogous results for the honest signal scenario are shown in Supplementary Figure S6. Other parameters take their default values (Table 1): in particular, $c_{L}=1;V=0.7;\theta_{B}=1.5;\theta_{S}=0;\delta_{F}=1;\delta_{W}=1;c_{C}=0.3$

Unsurprisingly, the equilibrium sizes of both honest and dishonest signals decreased as the cost $c_{S}$ of producing such signals increased (Figure 8A for dishonest signals; Supplementary Figure S6A for honest signals). On the other hand, investment in signal size increased if males were better at estimating perceived fighting ability in the display stage (i.e., larger $\delta_{C}$: Figure 8B for dishonest signals; Supplementary Figure S6B for honest signals). This is because larger signals are perceived more readily (i.e., with less noise) when $\delta_{C}$ is larger, making them more effective tools for intimidating opponents. Honest signals were similarly larger when actual fighting ability was perceived more accurately in the contact stage (i.e., larger $\delta_{F}$; Supplementary Figure S7A). In contrast, dishonest signals decreased slightly with increasing $\delta_{F}$ (Supplementary Figure S7B). Since dishonest signals played no role in the contact stage, the precision with which males can estimate actual fighting ability had no substantial effect on the size of these signals.

## Discussion

We have shown that signals that are displayed in the context of aggressive interactions can become exaggerated far beyond their ecological optima (Figure 5A and B). Investment in honest signals, which are directly linked to actual fighting ability, was predicted to be greater than investment in dishonest signals. Investment in dishonest signals was predicted to be highest when most aggressive interactions are resolved in the display stage, a result that could be tested empirically. In contrast to Moore et al. (2022), we found no evidence for runaway evolution of honest signals. Instead, such signals always evolved toward a stable equilibrium.

Our model differs in structure from that of Moore et al. (2022) in several respects. Most importantly, our model assumes that (a) deviating from the ecologically optimal body size is costly, (b) an individual’s probability of winning a fight increases with their body size, and (c) body size affects an individual’s perceived fighting ability and therefore the probability that an opponent escalates aggressive interactions. Body size thus affects fitness via three distinct causal pathways in our model. In contrast, body size is causally inert in the model of Moore et al. (2022), where it evolves purely due to genetic correlations with the aggressive signal. In our model, we assume no genetic correlation between body and signal size, although there is a mutational correlation between body size and perceived fighting ability. If all three of the above causal pathways (a–c) are disabled in our model, then body size is selectively neutral, and so, in the absence of genetic correlation, body size will not evolve. If body size is assumed to be costly but not to affect perceived or actual fighting ability, then it evolves to its ecologically optimal value and remains there. Lastly, if body size is allowed to affect perceived and/or actual fighting ability but is not costly, then body size approaches infinity over evolutionary time. This last outcome broadly resembles the runaway outcome predicted by Moore et al. (2022).

We assume that dishonest aggressive signals are not perceived by opponents directly, but rather via “perceived fighting ability,” which is determined by both body size and signal size (Figure 2). Since both perceived and actual fighting ability are functions of body size, an individual’s perceived fighting ability is a partially honest signal of their actual fighting ability. As a consequence, opponents have no mechanism to ignore dishonest signals without forgoing relevant, albeit imperfect, information about their opponent’s fighting ability. Therefore, there is always some advantage to developing such aggressive signals. Many empirical examples of dishonest signals arise similarly via the exaggeration of existing reliable cues. For example, in the dance-fly *Rhamphomyia longicauda*, males provide their mates with captured prey items as nuptial gifts (Funk & Tallamy, 2000). Since female body size correlates with fecundity, larger females are more attractive to males and hence more likely to benefit from their nuptial gifts. In response, females have evolved to exaggerate their body size using inflatable pouches on the pleural margins of their abdomens (Funk & Tallamy, 2000). Theory predicts that such dishonest exaggeration can be maintained as long as there remains a positive relationship between the trait of interest to the receiver (e.g., actual fighting ability in our model) and the signal as perceived by the receiver (e.g., perceived fighting ability in our model) (Biernaskie et al., 2018). In addition to the exaggeration of existing cues, such positive correlations can also be maintained if the trait of interest is quality dependent and if high-quality individuals can afford to spend more on signals than low-quality individuals (the “handicap principle”; Biernaskie et al., 2018; Fromhage & Henshaw, 2022; Grafen, 1990).

In our multistage model, as the costs of losing a fight increase, contestants lose interest in fighting and are thus more willing to engage in low-intensity physical contact in which actual fighting ability is assessed (Figure 7). As a consequence, a subset of contests can be resolved without costly physical fights. This is in line with numerous empirical studies, demonstrating that animal contests can end without a violent fight (e.g., Bentley et al., 2009; Camerlink et al., 2016). Our model predicts the average level of aggressiveness in populations. However, empirical studies conducted on a wide variety of species have shown that there are discernible and consistent differences in aggressiveness between individuals and that aggressiveness can even influence fighting ability, with bold individuals being more likely to win contests, though the opposite pattern is also possible (reviewed in Briffa et al., 2015). Therefore, incorporating individual variation into the model and allowing for correlations between body size, signal size, aggressiveness, and fighting ability may offer new insights into the evolution of honest and dishonest signals in future studies.

Although individuals can adjust their mean level of aggressiveness in our model, the shape of the reaction norms relating escalation probabilities to perceived differences in relative fighting ability is assumed fixed (i.e., $\delta_{C}$ and $\delta_{F}$ are fixed parameters). Future research could extend the model to allow for the steepness of these reaction norms to evolve (e.g., by modeling $\delta_{C}$ and $\delta_{F}$ as evolving traits, rather than fixed parameters). In the display stage, we might expect a nearly step-shaped reaction norm (cf. black line in Figure 4A) in populations where perceived fighting ability is strongly correlated with actual fighting ability, but a shallower gradient (cf. light gray line in Figure 4A) when this correlation is weak. If signal reliability is extremely low, we might even expect receivers to stop paying attention to the signal entirely (i.e., a completely flat reaction norm) (Bond, 1989; Dawkins & Guilford, 1991; Wiley, 1983). Nonetheless, even a weak correlation between perceived and actual fighting ability may be sufficient for the evolutionary maintenance of receiver responses, particularly if no other more reliable signals are available. For instance, Blanchard’s cricket frogs rely on calling frequency as a cue of fighting ability, even though the correlation between this signal and fighting ability is rather weak (Wagner, 1989a; Wagner, 1989b, Wagner, 1992). In modeling optimal receiver responses, it may also be important to account for the costs of evaluating signal reliability (e.g., in terms of time or cognitive effort) (Dawkins & Guilford, 1991; McNamara & Leimar, 2020).

Our model treats fitness costs and benefits as fixed parameters, rather than as emergent properties of a complete life-history model. A more elaborate model could consider how such costs and benefits emerge from the totality of aggressive and mating interactions over an individual’s lifetime, viewed in the context of an explicitly modeled resident population (Awasthi & Henshaw, 2023; Houston & McNamara, 2005; McNamara & Leimar, 2020). Such a model could allow for eco-evolutionary interactions and feedbacks that are not considered in our current model. Importantly, a individual’s optimal level of aggression in any given interaction should depend not only on the contestants’ traits but also on the broader population context. In particular, how easy is it to locate resources, and how strong is competition for such resources typically? Both factors depend crucially on population density and the extent to which resources are aggregated in space (Emlen & Oring, 1977). Furthermore, high rates of injury or mortality due to fights should remove affected individuals from the pool of competitors, leading to a relaxation of competition and more uncontested resources (i.e., negative feedback). In the case of male–male competition for females, this would manifest itself as a female-biased adult sex ratio (Emlen & Oring, 1977). Future research could attempt to gain a deeper understanding of such interactions by examining how population density and the spatial aggregations of resources shape the evolution of aggression, aggressive signals, and weaponry (Knell, 2009). Two further limitations are worth pointing out. First, to ease comparison, we assume that honest and dishonest signals are similarly costly; in reality, however, dishonest signals may often provide a cheaper means to intimidate opponents than honest signals. This could favor relatively greater investment in dishonest signals when individuals can choose which types of signal to invest in. More generally, our model treats honest and dishonest signals as fixed categories, but future work could allow the honesty level of a signal to evolve dynamically alongside its size (see also Biernaskie et al., 2018; Fromhage & Henshaw, 2022).

## Supplementary Material

qrae008_suppl_Supplementary_Material

## Data Availability

Wolfram Mathematica code for the adaptive dynamic simulations is available in Supplementary Materials.
