# Supplementary material for: The evolution of honest and dishonest signals of fighting ability
Source: Evol Lett. 2024 Mar 8;8(4):514–25. doi: 10.1093/evlett/qrae008 (PMC11497847; doi:10.1093/evlett/qrae008)
Supplement: qrae008_suppl_Supplementary_Material [file qrae008_suppl_supplementary_material.zip › Supplementary__The_evolution_of_honest_and_dishonest_signals_of_fighting_ability.pdf]

Supplementary Materials: The evolution of honest and dishonest signals of fighting ability

MOHAMMADALI DASHTBALI, XIAOYAN LONG AND JONATHAN M. HENSHAW

Causal analyses

To understand how parameter values – in particular, the cost of losing a fight c_L – shape equilibrium trait values, we conducted formal causal analyses. Similar methods are used in econometrics under the banner of 'comparative statics' [1, 2]. The four traits in our model (i.e., g_B, g_S, α_C and α_F) potentially coevolve in response to changes in parameters. The first step is consequently to understand the direction of this coevolution: Which traits affect the selection environment for which other traits? To do this, let us consider the fitness function for our model, which is given by Equation (10) and further unpacked in Equations (9), (14-15), and (18-19) in the main text. Body size (g_B or g'_B) occurs in this fitness function in two forms: First, as part of the cost function $c(g_B, g_S)$ (Equation 7), and second, in ratios of body sizes B'/B, which show up in expressions for relative fighting ability (F'_P/F_P or F'_A/F_A) and thereby in the probabilities of escalating interactions (P_C, P'_C, P_F, P'_F) or winning fights (P_W, P'_W). The derivatives of the cost function with respect to g_S, α_C and α_F are independent of both mutant and resident body size. Consequently, resident body size cannot shape selection on the other traits via the cost function. Moreover, since the mean and variance of body size are proportional to g_B (Equation 3), the distribution of the ratio B'/B is also independent of resident body size when evaluated at $g_B = g'_B$. The selection gradients on g_S, α_C and α_F (Equation 21) are therefore independent of body size. On the other hand, coevolutionary feedback between g_S, α_C and α_F is possible. The coevolutionary relationships between these traits under variation in c_L are summarized in Figure S1.

We can quantify the coevolutionary feedback among these traits as follows. First, let us write $g_S(c_L)$ for the equilibrium value of mean signal size, viewed as a function of c_L, with the values of all other parameters held fixed at some arbitrary values. This equilibrium results from the coevolution of g_S with the other three traits (α_C, α_F, and g_B). By considering nearby values of c_L, we can estimate numerically how steeply g_S changes with the cost of losing:

$$\frac{\delta g_S(c_L)}{\delta c_L} \approx \frac{g_S(c_L + \varepsilon) - g_S(c_L)}{\varepsilon}. \tag{S1}$$

This *causal derivative* [3, 4] measures how g_S changes with c_L while keeping all other parameters fixed, but allowing the other traits to vary due to the change in c_L and coevolutionary feedback among traits (Figure S1). To understand the causal mechanisms by which c_L affects g_S, we can then selectively disable some of these causal pathways. For example, to understand the direct effect of c_L on g_S, we can hold both α_C and α_F constant. Writing $\alpha_C(c_L)$ and $\alpha_F(c_L)$ for the (natural) equilibrium values of these variables at c_L, the expression $g_S(c_L + \varepsilon)\Big|_{\substack{\alpha_C = \alpha_C(c_L), \\ \alpha_F = \alpha_F(c_L)}}$ indicates the equilibrium value of g_S at $c_L + \varepsilon$ when α_C and α_F are held fixed at their equilibrium values at c_L. In practice, this equilibrium can be obtained by iterating Equation (22) while resetting $\alpha_C = \alpha_C(c_L)$ and $\alpha_F = \alpha_F(c_L)$ after each iteration. The rate of change in g_S due solely to the direct effect $c_L \to g_S$ is then given by the *path-specific* causal derivative:

$$\left.\frac{\delta g_S(c_L)}{\delta c_L}\right|_{c_L \to g_S} \approx \frac{g_S(c_L + \varepsilon)\Big|_{\substack{\alpha_C = \alpha_C(c_L), \\ \alpha_F = \alpha_F(c_L)}} - g_S(c_L)}{\varepsilon}. \tag{S2}$$

Similar path-specific derivatives can be defined to test hypotheses about the coevolutionary relationships among other variables. When interpreting figures involving such gradients (Figures

S4 and S5), it is important to realize that these gradients are first derivatives. Thus, a positive gradient indicates an increasing relationship between the trait and the parameter (e.g., c_L), whereas a negative gradient indicates a decreasing relationship. We note the following patterns, which support the verbal explanations in the main text (please note that all causal analyses are based on the range of c_L values represented by the right halves of Figures 5 and 7 in the main text):

- **Willingness to escalate to physical contact,** α_C: When α_F is held fixed, equilibrium α_C consistently decreases with c_L (Figure S4A for the dishonest signal scenario, Figure S5A for the honest signal scenario). This contrasts with the natural patterns, whereby equilibrium α_C first decreases and then increases with c_L (Figure 7 in main text for the dishonest signal scenario; Figure S3 for the honest signal scenario). This indicates that the direct effect of c_L on α_C is negative, whereas the indirect effect via α_F is positive (please note that the effects of g_S on α_C and α_F are very weak; results not shown).

- **Willingness to escalate to a full fight,** α_F: In contrast, the effect of α_C on α_F is very weak: If we hold α_C constant, then the equilibrium value of α_F barely deviates from its natural equilibrium (Figure S4B and S5B).

- **Dishonest signals**: When both α_C and α_F are artificially held fixed, dishonest signals stay constant as c_L increases (dotted line in Figure S4C). This indicates that c_L has no strong direct effect on the size of dishonest signals. Similarly, if only α_C is held fixed and α_F is allowed to evolve, then dishonest signals again stay constant with c_L (square markers in Figure S4C). In contrast, if only α_F is held fixed, so that α_C declines with c_L (see above), then dishonest signals grow in size with increasing c_L (circle markers in Figure S4C). This indicates that the effect of c_L on dishonest signals operates indirectly via α_C: The more willing individuals are to escalate to physical contact, the smaller dishonest signals should be at equilibrium. Consistent with this, when all traits are allowed to coevolve, α_C increases with c_L (black line in Figure S4A) and dishonest signals decrease in size (triangle markers in Figure S4C).

- **Honest signals**: When α_C and α_F are held artificially fixed, honest signals grow in size with c_L (dash-dotted line in Figure S5C). The direct effect of c_L on honest signals is consequently positive. If α_F is held fixed, but α_C is allowed to vary, then α_C decreases with c_L (grey line in Figure S5A) and so does honest signal size (circle markers in Figure S5C), indicating that α_C has a positive effect on the size of honest signals. Similar logic applies to α_F. Hence, c_L affects honest signal size both directly and indirectly via α_C and α_F. The patterns for body size are very similar (results not shown).

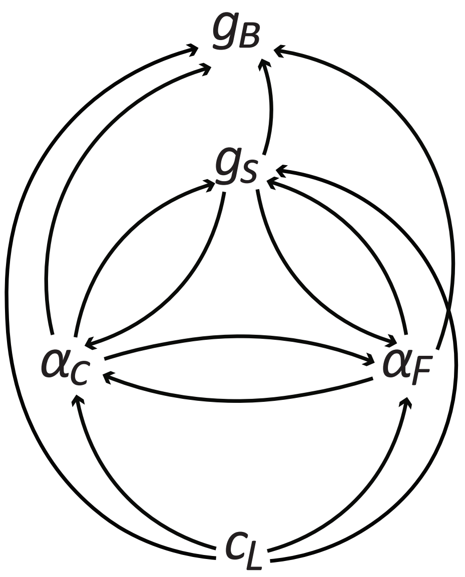

Fig. S1. Causal relationships between the cost of losing a fight, c_L, and the equilibrium values of the tendency to escalate to contact, α_C, the tendency to escalate to a fight, α_F, and the breeding value for signal size, g_S, and body size, g_B. Arrows of the form $A \rightarrow B$ indicate a causal effect of A on B.

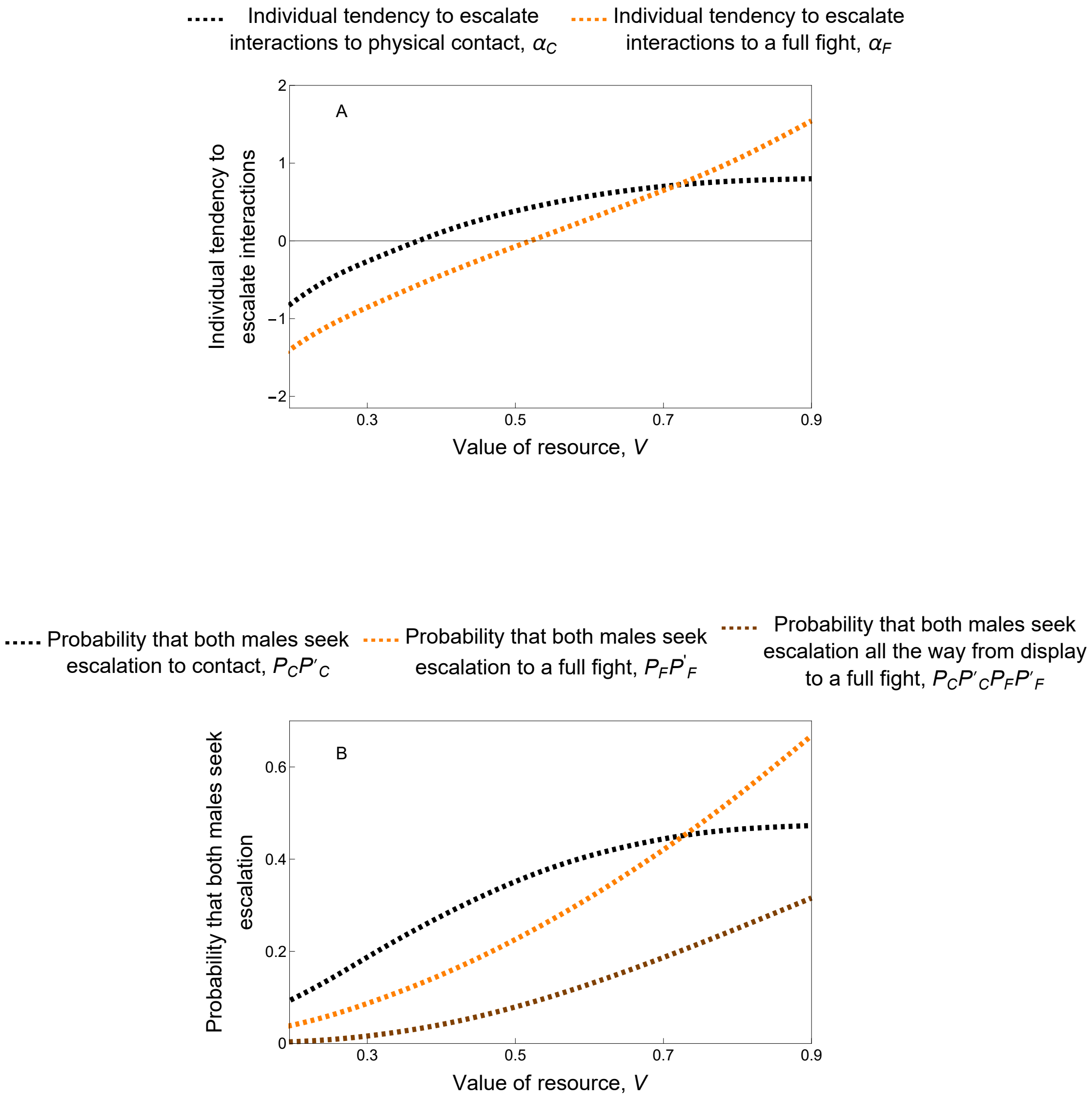

Fig. S2. (A) Equilibrium tendency to escalate interactions to physical contact, α_C (black dashed) or to a full fight, α_F (orange dashed) for the honest signal scenario with respect to the value of resource. (B) The probability that a pair of males escalates the interaction from the display stage to physical contact, $P_C P'_C$ (black dashed), from the contact stage to a full fight, $P_F P'_F$ (orange dashed), or from the display stage all the way to a full fight, $P_C P'_C P_F P'_F$ (brown dashed) for the honest signal scenario, averaging over all environmental effects. Other parameters take their default values (Table 1): in particular, $c_L = 1$; $\theta_B = 1.5$; $\theta_S = 0$; $\delta_C = 0.5$; $\delta_F = 1$; $\delta_W = 1$; $c_S = 0.03$; $c_C = 0.3$.

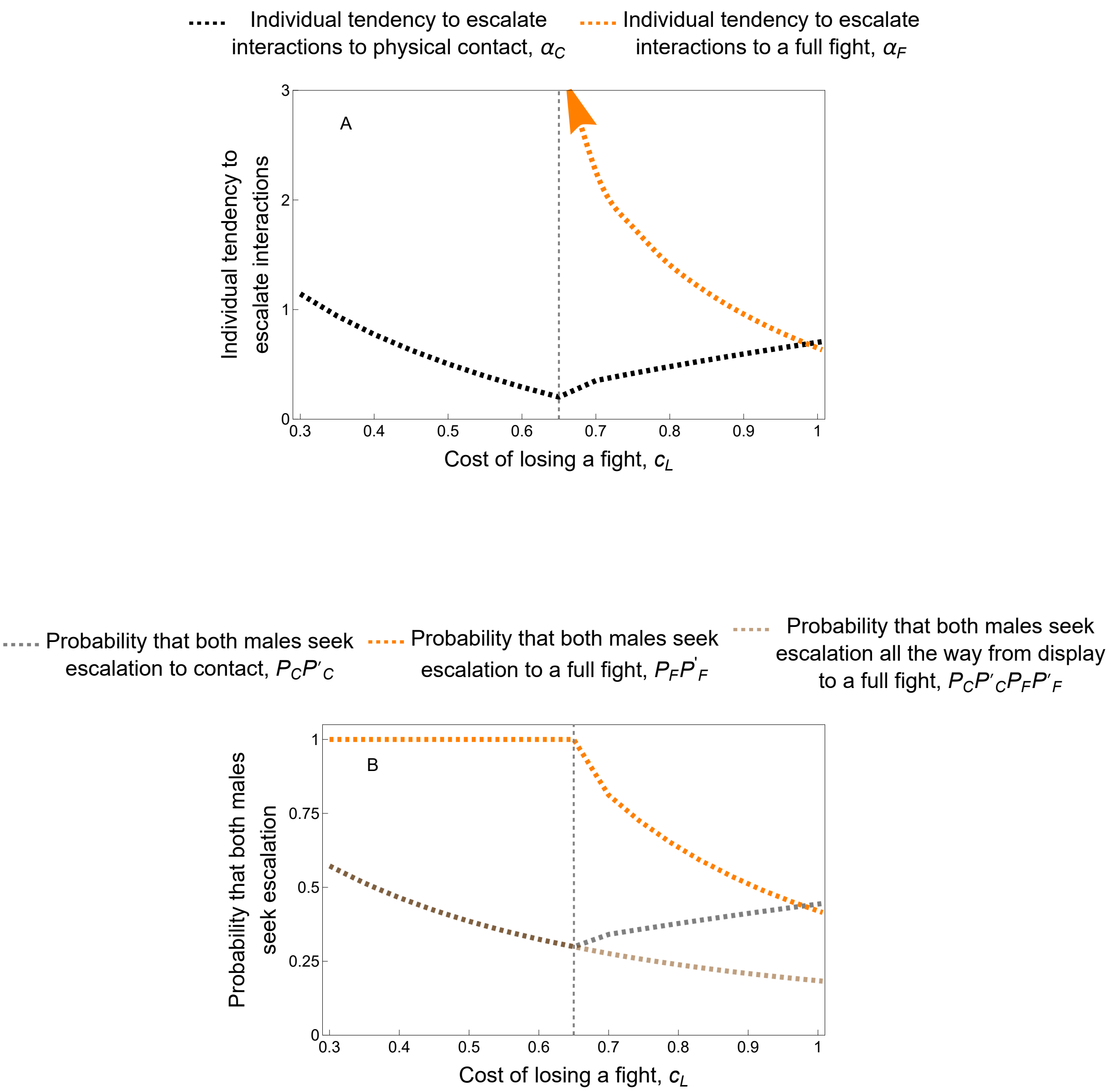

Fig. S3. (A) Equilibrium tendency to escalate interactions to physical contact, α_C (black dashed) or to a full fight, α_F (orange dashed) for the honest signal scenario with respect to the cost of losing a fight. (B) The probability that a pair of males escalates the interaction from the display stage to physical contact, $P_C P'_C$ (grey dashed), from the contact stage to a full fight, $P_F P'_F$ (orange dashed), or from the display stage all the way to a full fight, $P_C P'_C P_F P'_F$ (brown dashed) for the honest signal scenario, averaging over all environmental effects. The vertical lines correspond to the c_L value indicated by the vertical lines in Figure 5A,C. Other parameters take their default values (Table 1): in particular, $V = 0.7$; $\theta_B = 1.5$; $\theta_S = 0$; $\delta_C = 0.5$; $\delta_F = 1$; $\delta_W = 1$; $c_S = 0.03$; $c_C = 0.3$. For $c_L > V$, we used initial values as in Table 1. For $c_L < V$, convergence was very slow. To speed up convergence in this parameter range, we consequently took the equilibrium trait values for each c_L-value as initial values for the next-smallest c_L-value.

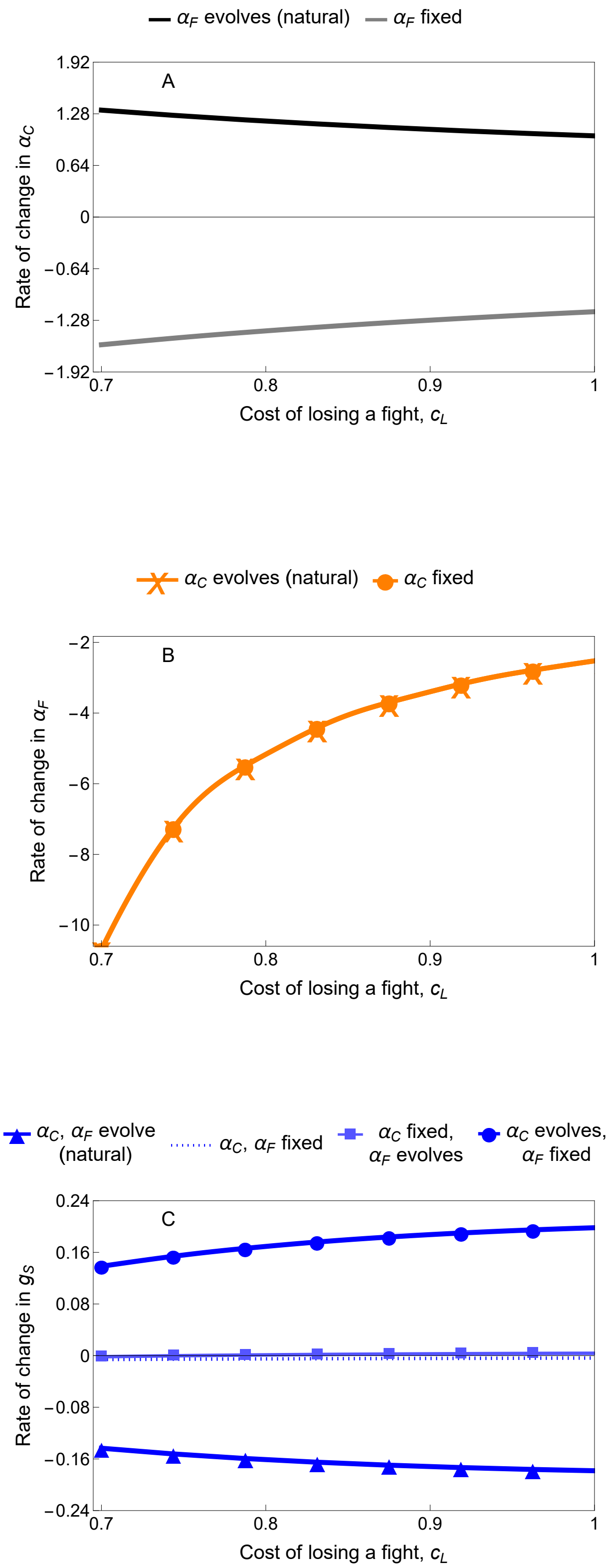

Fig. S4. Rates of change in (A) the equilibrium tendency to escalate to physical contact α_C, (B) the equilibrium tendency to escalate to a full fight α_F and (C) the equilibrium signal size g_S with respect to the cost of losing a fight, c_L for the dishonest signal scenario. Shown are both the natural gradients and the causal gradients obtained by holding fixed a subset of the (normally coevolving) traits (see inset legends). Other parameters take their default values (Table 1): in particular, $V = 0.7$; $\theta_B = 1.5$; $\theta_S = 0$; $\delta_C = 0.5$; $\delta_F = 1$; $\delta_W = 1$; $c_S = 0.03$; $c_C = 0.3$.

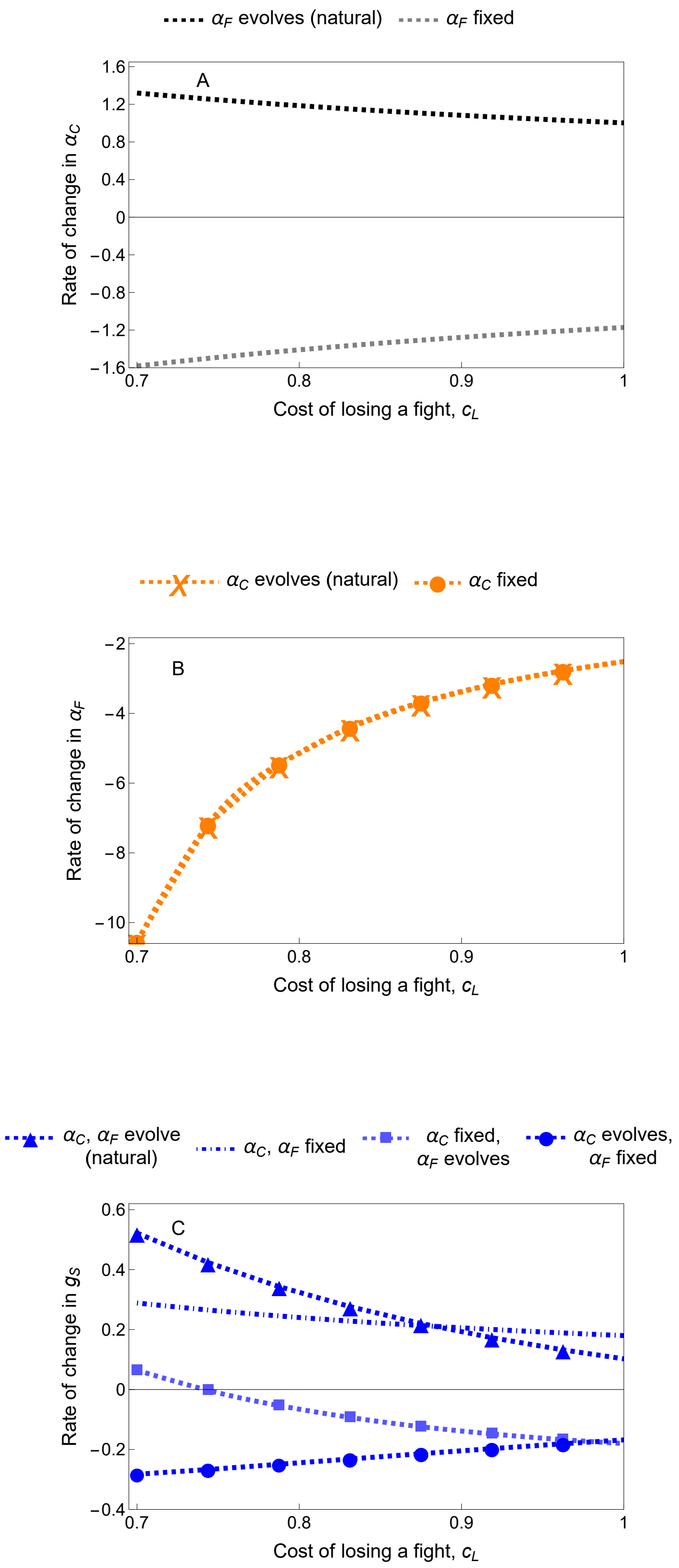

Fig. S5. Rates of change in (A) the equilibrium tendency to escalate to physical contact α_C, (B) the equilibrium tendency to escalate to a full fight α_F and (C) the equilibrium signal size g_S with respect to the cost of losing a fight, c_L for the honest signal scenario. Shown are both the natural gradients and the causal gradients obtained by holding fixed a subset of the (normally coevolving) traits (see inset legends). Other parameters take their default values (Table 1): in particular, $V = 0.7$; $\theta_B = 1.5$; $\theta_S = 0$; $\delta_C = 0.5$; $\delta_F = 1$; $\delta_W = 1$; $c_S = 0.03$; $c_C = 0.3$.

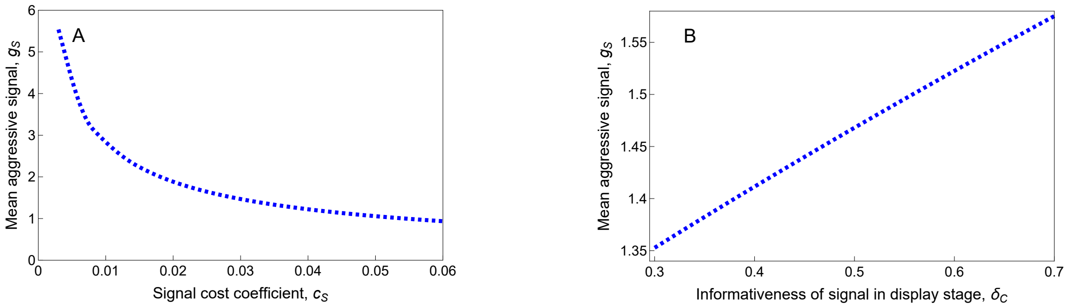

Fig. S6. Equilibrium mean signal size with respect to (A) the signal cost coefficient, c_S (shown with $c_L = 1;\ \delta_C = 0.5$), and (B) the informativeness of signal in display stage, δ_C (shown with $c_L = 1,\ c_S = 0.03$) for the honest signal scenario. Other parameters take their default values (Table 1): in particular, $V = 0.7;\ \theta_B = 1.5;\ \theta_S = 0;\ \delta_F = 1;\ \delta_W = 1;\ c_C = 0.3$.

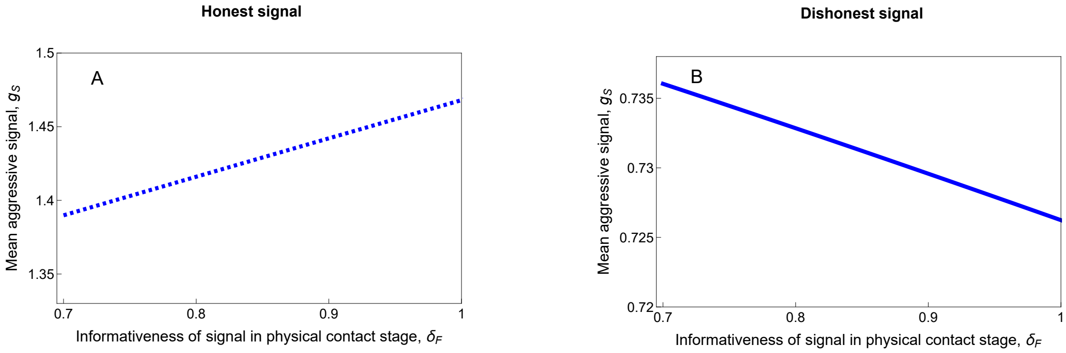

Fig. S7. Equilibrium mean signal size with respect to the informativeness of signals in the physical contact stage, δ_F for (A) honest signals and (B) dishonest signals. Other parameters take their default values (Table 1): in particular, $c_L = 1;\ V = 0.7;\ \theta_B = 1.5;\ \theta_S = 0;\ \delta_C = 0.5;\ \delta_W = 1;\ c_C = 0.3$.

REFERENCES

1. Acemoglu, D. & Jensen, M.K., 2013. Aggregate comparative statics. Games and Economic Behavior, 81, pp.27-49.
2. Cunningham, S., 2021. Causal inference: The mixtape. New Haven: Yale University Press.
3. Henshaw, J. M., Morrissey, M. B. and Jones, A. G., (2020). Quantifying the causal pathways contributing to natural selection. Evolution, 74(12), pp.2560-2574.
4. Henshaw, J.M., Fromhage, L. and Jones, A.G.,2022. The evolution of mating preferences for genetic attractiveness and quality in the presence of sensory bias. Proceedings of the National Academy of Sciences of the USA, 119(33), p.e2206262119.
